# Rapid Interval Development of a Left Hepatic Artery Pseudoaneurysm During Acute Interstitial Edematous Pancreatitis

**DOI:** 10.7759/cureus.111943

**Published:** 2026-07-02

**Authors:** Balakrishna Ravella, Abhinay Theli, Eswar Chand Gadde, Harish Ravipati

**Affiliations:** 1 Internal Medicine, OSF Health St. Mary Medical Center, Galesburg, USA; 2 Medicine, Guthrie Cortland Medical Center, Cortland, USA; 3 Internal Medicine, Ascension St. John Hospital, Tulsa, USA; 4 Internal Medicine, Guthrie Cortland Medical Center, Cortland, USA

**Keywords:** acute pancreatitis, hepatic artery pseudoaneurysm, interstitial edematous pancreatitis, intra-abdominal hemorrhage, vascular complication

## Abstract

Visceral artery pseudoaneurysms are uncommon but potentially life-threatening vascular complications of acute pancreatitis. They are most frequently reported in association with necrotizing or chronic pancreatitis and typically develop weeks after disease onset. Hepatic artery pseudoaneurysms are particularly rare.

We report a 70-year-old man with presumed idiopathic acute interstitial edematous pancreatitis whose serial contrast-enhanced computed tomography (CT) examinations documented interval development of a left hepatic artery pseudoaneurysm within four days of presentation despite the absence of pancreatic necrosis, pseudocyst formation, or walled-off necrosis. Initial imaging demonstrated uncomplicated interstitial edematous pancreatitis without vascular abnormalities. Repeat imaging obtained because of worsening abdominal pain and abdominal distension revealed a new 2.3 × 2.0 cm enhancing lesion contiguous with the left hepatic artery, highly suspicious for pseudoaneurysm. Shortly after imaging, the patient developed pulseless electrical activity cardiac arrest and died despite prolonged resuscitative efforts.

This case broadens the recognized spectrum of pancreatitis-associated vascular complications by demonstrating rapid interval development of a left hepatic artery pseudoaneurysm during acute interstitial edematous pancreatitis without pancreatic necrosis. It also highlights the importance of repeat vascular assessment, preferably with dedicated arterial-phase imaging when feasible, in patients with worsening symptoms or unexpected clinical deterioration.

## Introduction

Acute pancreatitis remains one of the most common gastrointestinal causes of hospitalization in the United States, with an annual incidence of approximately 13-45 cases per 100,000 persons [1]. Most cases are mild and self-limited; however, 15%-20% progress to moderately severe or severe disease characterized by local complications or organ failure [2]. According to the Revised Atlanta Classification, acute pancreatitis is morphologically classified as either interstitial edematous pancreatitis or necrotizing pancreatitis. Interstitial edematous pancreatitis is characterized by diffuse pancreatic enlargement and homogeneous enhancement without parenchymal necrosis, whereas necrotizing pancreatitis involves pancreatic and/or peripancreatic tissue necrosis and carries a substantially higher risk of local vascular complications [3].

Vascular complications of pancreatitis, particularly visceral artery pseudoaneurysms, are uncommon but carry substantial morbidity and mortality. Pseudoaneurysms develop when pancreatic proteolytic enzymes and surrounding inflammation erode the arterial wall, resulting in disruption of the intima and media and the formation of a contained arterial rupture bounded only by surrounding tissues rather than by a true vessel wall [4]. The splenic and gastroduodenal arteries are most frequently involved; hepatic artery pseudoaneurysms (HAPs) represent only a small minority of reported cases [5,6]. When rupture occurs, mortality can exceed 50%-90% without prompt intervention [7,8].

Although pseudoaneurysms are typically described in necrotizing or chronic pancreatitis and often develop weeks after disease onset, their early occurrence in interstitial edematous pancreatitis appears to be exceedingly uncommon. To better characterize the available literature, we performed a focused search of PubMed and Google Scholar through April 2026 using combinations of the terms "hepatic artery pseudoaneurysm," "left hepatic artery pseudoaneurysm," "acute pancreatitis," "interstitial edematous pancreatitis," and "vascular complications of pancreatitis." The available literature consisted primarily of isolated case reports and small case series, with very few reports documenting serial imaging that clearly demonstrated interval pseudoaneurysm formation during the early course of interstitial edematous pancreatitis, most often following endoscopic interventions or in the setting of chronic disease [9,10].

We therefore present a case of presumed idiopathic acute interstitial edematous pancreatitis in which serial contrast-enhanced CT examinations documented rapid interval development of a left hepatic artery pseudoaneurysm within four days of presentation. This case expands the recognized spectrum of vascular complications associated with interstitial edematous pancreatitis and highlights the importance of repeat vascular assessment in patients who experience worsening abdominal pain or unexpected clinical deterioration.

## Case presentation

A 70-year-old man with a history of primary hypertension, obstructive sleep apnea, and stage IV metastatic prostate adenocarcinoma (Gleason 7, Grade Group 3, T3bN0M1) status post prostatectomy and adjuvant radiation, receiving leuprolide every three months, darolutamide 300 mg twice daily, and monthly denosumab injections, presented to the emergency department with two days of midepigastric abdominal pain radiating to back, nausea, and vomiting. He denied alcohol use, prior episodes of pancreatitis, recent endoscopic retrograde cholangiopancreatography (ERCP), abdominal trauma, or recent medication changes. Although the patient had metastatic prostate cancer, there was no clinical or radiographic evidence of pancreatic involvement or biliary obstruction. None of his current medications (Table 1) are commonly associated with acute pancreatitis.

**Table 1:** List of medications This table shows the patient’s home medication list at the time of admission.

Medication	Dose	Frequency
Cetirizine	10 mg	Daily
Cyanocobalamin	1000 mcg	Daily
Darolutamide	300 mg	Twice daily
Denosumab (Xgeva)	120 mg subcutaneous	Every 30 days
Ferrous sulfate	325 mg	Daily
Lisinopril	30 mg	Daily
Multivitamin	1 tablet	Daily
Leuprolide	22.5 mg intramuscular	Every 3 months

Vital signs on admission were temperature 98.5°F, heart rate 110/min, blood pressure 157/97 mmHg, respirations 14/min, and oxygen saturation 93% on room air. The physical examination revealed a soft abdomen with midepigastric tenderness without rebound or guarding. Laboratory evaluation was significant for leukocytosis (white blood cell count, 18.75 × 10³/µL, with 91.2% neutrophils), lipase 1138 U/L, blood urea nitrogen 45 mg/dL, creatinine 1.91 mg/dL, glucose 177 mg/dL, and total bilirubin 1.5 mg/dL (Table 2). Triglycerides were normal (107 mg/dL).

**Table 2:** Laboratory values Serial laboratory values obtained on hospital day 1 (admission), day 2, day 3, and day 4 (during cardiac arrest), demonstrating an acute hemoglobin drop.

Laboratory parameters	Reference range	Day 1 (Admission)	Day 2	Day 3	Day 4/During arrest
White blood cell count (×10³/µL)	4.0–10.0	18.75	13.85	11.62	9.56
Hemoglobin (g/dL)	13.5–17.5	14.8	12.3	12.5	7.1
Platelet count (×10³/µL)	150–400	371	247	217	275
Sodium (mEq/L)	135–145	136	140	130	152
Potassium (mEq/L)	3.5–5.0	4.8	4.2	3.5	5.4
Blood urea nitrogen (mg/dL)	7–20	45	26	15	17
Creatinine (mg/dL)	0.6–1.2	1.91	1.12	0.83	1.25
Calcium (mg/dL)	8.5–10.5	10.5	7.4	6.8	8.4
Aspartate aminotransferase (U/L)	10–40	31	29	33	58
Alanine aminotransferase (U/L)	7–56	21	14	15	34
Alkaline phosphatase (U/L)	40–150	49	33	35	67
Total bilirubin (mg/dL)	0.3–1.2	1.1	0.9	0.8	0.5

Contrast-enhanced computed tomography (CT) scan of the abdomen and pelvis revealed extensive peripancreatic fluid and fat stranding consistent with acute interstitial edematous pancreatitis; the pancreas enhanced homogeneously (Figure 1). Right upper quadrant ultrasound showed a distended gallbladder without calculi or sludge, minimal pericholecystic fluid (Figure 1), a negative sonographic Murphy sign, and a common bile duct measuring 4.6 mm without choledocholithiasis. Based on the characteristic clinical presentation, elevated serum lipase, and imaging findings, the patient fulfilled the diagnostic criteria for acute interstitial edematous pancreatitis. Alternative etiologies were systematically evaluated. Gallstone pancreatitis was considered unlikely because ultrasonography demonstrated no cholelithiasis or biliary ductal dilation. Normal triglyceride levels excluded hypertriglyceridemia; the patient denied alcohol use; there had been no recent ERCP or abdominal trauma, and no medication was identified as a likely precipitating factor. In the absence of an identifiable etiology, the episode was classified as presumed idiopathic acute interstitial edematous pancreatitis. The clinical course met Revised Atlanta criteria for moderately severe acute pancreatitis because of transient organ dysfunction without persistent organ failure or pancreatic necrosis.

**Figure 1:**
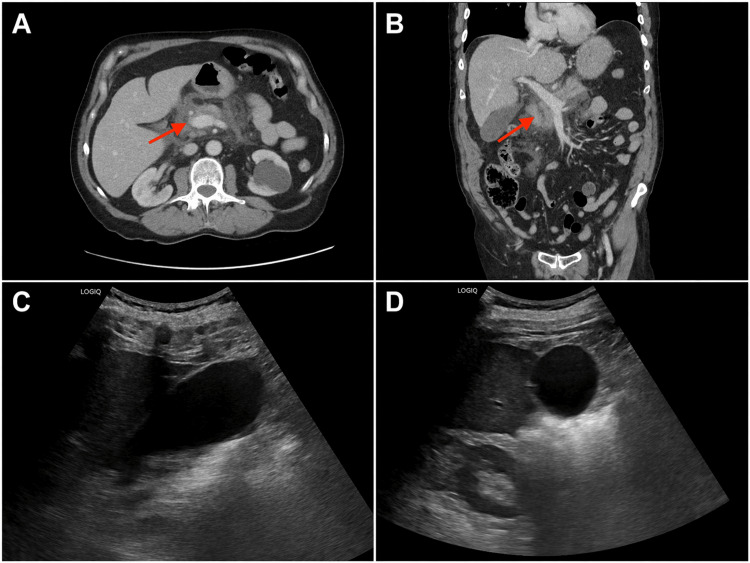
Computed tomography scan of the abdomen and pelvis and right upper quadrant ultrasound on day 1 of admission (A) and (B) Axial and coronal views of the CT abdomen and pelvis showing peripancreatic fluid and fat stranding consistent with acute pancreatitis. (C) and (D) Longitudinal and transverse views of the gall bladder showing a distended gall bladder without any calculi or sludge and with minimal pericholecystic fluid.

The patient was admitted, made nil per os, and started on aggressive intravenous fluid resuscitation with lactated Ringer's solution at 150 mL/h and intravenous hydromorphone as needed for pain control. On hospital day 2, his abdominal pain improved, and he tolerated a clear liquid diet; intravenous fluids were subsequently discontinued. On hospital day 3, he developed recurrent nausea and vomiting accompanied by progressively worsening epigastric pain, requiring escalation to hydromorphone patient-controlled analgesia (PCA). Repeat contrast-enhanced CT demonstrated interval progression of peripancreatic inflammatory changes with increased peripancreatic fluid, diffuse pancreatic edema, new small-volume intraperitoneal free fluid, bilateral pleural effusions, and bibasilar atelectatic changes (Figure 2). Importantly, there remained no radiographic evidence of pancreatic necrosis, pseudocyst formation, walled-off necrosis, or vascular abnormality.

**Figure 2:**
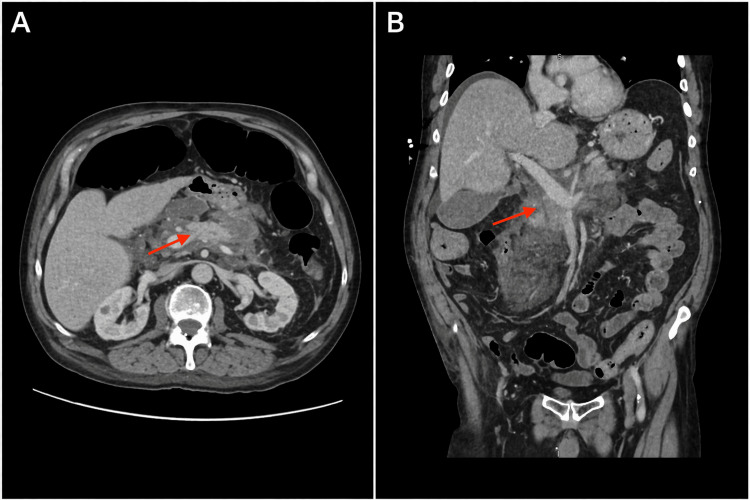
Contrast-enhanced computed tomography of the abdomen and pelvis obtained on hospital day 3 (A) Axial and (B) coronal images demonstrate interval progression of acute interstitial edematous pancreatitis with increased peripancreatic inflammatory stranding, diffuse pancreatic edema, and increased peripancreatic fluid collections. No pancreatic necrosis or vascular abnormality is identified.

By hospital day 4, despite PCA analgesia, abdominal pain had further intensified and was accompanied by progressive abdominal distension and new voluntary guarding, representing an unexpected deviation from the anticipated clinical course of uncomplicated interstitial edematous pancreatitis. Repeat contrast-enhanced CT of the abdomen and pelvis demonstrated a newly developed 2.3 × 2.0 cm rounded enhancing lesion within the left hepatic lobe that enhanced similarly to adjacent arterial structures and appeared contiguous with the expected course of the left hepatic artery, findings interpreted by the attending radiologist as highly suspicious for a left hepatic artery pseudoaneurysm (Figure 3). The lesion had not been present on either of the two previous contrast-enhanced CT examinations, documenting interval development during hospitalization. Because imaging was obtained during the portal venous phase rather than with dedicated CT angiography or an arterial-phase CT examination, definitive characterization of the lesion and its arterial neck was not possible.

**Figure 3:**
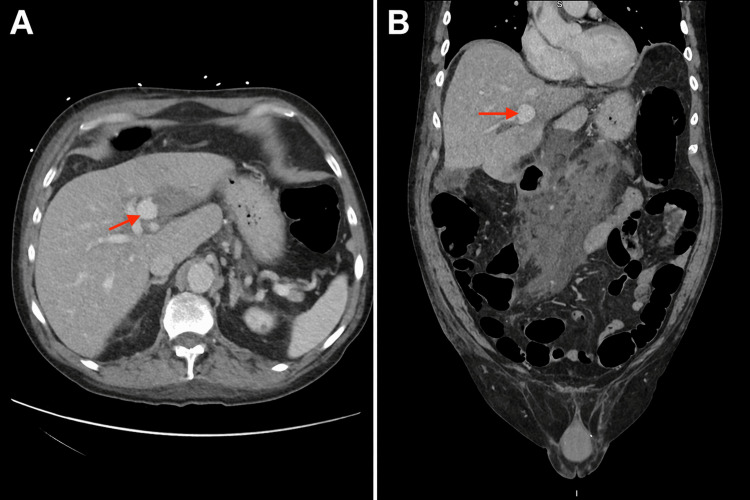
Contrast-enhanced computed tomography of the abdomen and pelvis obtained on hospital day 4 (A) Axial and (B) coronal images demonstrate a newly developed rounded enhancing lesion (red arrows) within the left hepatic lobe that demonstrates enhancement similar to adjacent arterial structures and is contiguous with the expected course of the left hepatic artery, highly suspicious for a left hepatic artery pseudoaneurysm. This lesion was absent on the two preceding CT examinations, documenting interval development during hospitalization.

Approximately 15 minutes after returning from CT imaging, the patient developed pulseless electrical activity cardiac arrest. Interventional radiology consultation for further vascular evaluation was being pursued; however, the patient's abrupt clinical deterioration precluded additional diagnostic evaluation or endovascular intervention. Advanced cardiovascular life support was performed for approximately 30 minutes without return of spontaneous circulation. Laboratory testing obtained during resuscitation demonstrated an acute decline in hemoglobin to 7.1 g/dL compared with prior measurements (Table 2). Although this decline raised concern for possible hemorrhage, it occurred during active resuscitation and therefore could also have been influenced by hemodilution associated with intravenous fluid administration. The family declined autopsy.

Because catheter angiography, operative exploration, CT angiography, and autopsy were not performed, the precise mechanism underlying the patient's sudden cardiovascular collapse could not be definitively established. Nevertheless, the temporal relationship between interval pseudoaneurysm formation, progressive abdominal symptoms, acute anemia, and subsequent pulseless electrical activity arrest raised concern for a clinically significant vascular complication; however, causality could not be established.

## Discussion

Visceral artery pseudoaneurysms represent one of the most feared vascular complications of acute pancreatitis, arising from autodigestion of the arterial wall by activated pancreatic enzymes, particularly elastase and trypsin [4,11]. This enzymatic injury leads to focal disruption of the intima and media, resulting in a contained rupture bounded only by surrounding inflammatory tissue rather than a true arterial wall. Compared with true aneurysms, pseudoaneurysms carry a substantially higher risk of rupture because of the absence of structural integrity.

The reported incidence of pseudoaneurysm formation in pancreatitis varies from 1% to 10%, with higher rates observed in necrotizing disease and chronic pancreatitis [2,6]. The splenic artery accounts for approximately 40%-60% of cases, followed by the gastroduodenal and pancreaticoduodenal arteries [4]. Hepatic artery involvement is rare and more commonly associated with hepatobiliary interventions, trauma, or liver transplantation rather than primary pancreatic inflammation [5,9].

The principal novelty of the present case lies not only in the uncommon vascular territory involved but also in the remarkably early interval development of the lesion. Serial contrast-enhanced CT examinations demonstrated the absence of any vascular abnormality on admission and again on hospital day 3, followed by the appearance of a new left hepatic artery pseudoaneurysm on hospital day 4. To our knowledge, very few published reports have documented such rapid interval formation of a left hepatic artery pseudoaneurysm during acute interstitial edematous pancreatitis using serial imaging.

Traditionally, clinically significant vascular complications have been regarded as late manifestations of pancreatitis, developing after prolonged exposure of adjacent vessels to pancreatic enzymes and inflammatory mediators, frequently in association with pancreatic necrosis, pseudocyst formation, or walled-off necrosis. In contrast, our patient demonstrated interval pseudoaneurysm formation despite the absence of pancreatic necrosis throughout hospitalization. This observation suggests that severe vascular injury may occasionally occur earlier in the disease course than traditionally appreciated and that pancreatic necrosis may not be an absolute prerequisite for arterial wall injury. One possible explanation is extension of proteolytic and inflammatory injury along the hepatoduodenal ligament, resulting in focal arterial wall weakening despite preservation of pancreatic parenchymal enhancement. Although this mechanism remains speculative, it is biologically plausible and has been proposed in previous reports describing HAPs complicating acute pancreatitis [5,8].

Clinically, pseudoaneurysms may remain silent until rupture or may present with nonspecific symptoms such as worsening abdominal pain, gastrointestinal bleeding, or unexplained anemia [7]. Our patient's progressive abdominal pain, increasing abdominal distension, and deviation from the anticipated clinical course prompted repeat imaging despite the absence of necrosis on previous examinations. These findings emphasize that unexpected clinical deterioration, rather than disease severity alone, should prompt reconsideration of the differential diagnosis and evaluation for vascular complications.

Diagnosis of visceral artery pseudoaneurysms relies primarily on contrast-enhanced CT angiography or catheter angiography. Although portal venous phase CT may identify an enhancing vascular lesion, dedicated arterial-phase imaging provides superior characterization of the arterial anatomy, delineation of the pseudoaneurysm neck, and detection of active contrast extravasation [8]. In the present case, repeat imaging was performed using a portal venous phase protocol because it reflected routine clinical care rather than dedicated vascular assessment. Consequently, although the lesion demonstrated arterial-equivalent enhancement and continuity with the expected course of the left hepatic artery, definitive angiographic confirmation was unavailable. This limitation has been acknowledged and should be considered when interpreting the findings.

Current evidence does not support routine repeat vascular imaging in all patients with acute interstitial edematous pancreatitis. However, our case suggests that repeat contrast-enhanced imaging, preferably with dedicated arterial-phase CT angiography when feasible, should be considered in patients who develop unexpected clinical deterioration, worsening abdominal pain, progressive abdominal distention, unexplained anemia, or other findings that are disproportionate to the anticipated clinical course. Early recognition of a pseudoaneurysm is clinically important because contemporary endovascular techniques, including coil embolization, covered stent placement, and liquid embolic agents, achieve high technical success rates and are considered first-line therapy in hemodynamically stable patients [12].

The patient's sudden pulseless electrical activity arrest occurred shortly after identification of the pseudoaneurysm, and the accompanying decline in hemoglobin raised concern for a possible hemorrhagic complication. Nevertheless, definitive evidence of pseudoaneurysm rupture was unavailable because catheter angiography, operative exploration, CT angiography, and autopsy were not performed. Furthermore, the hemoglobin measurement was obtained during active resuscitation, and hemodilution from intravenous fluid administration may have contributed to the observed decline. Alternative mechanisms of pulseless electrical activity arrest, including severe systemic inflammatory response, metabolic derangements, or other complications of acute pancreatitis, therefore cannot be excluded. Accordingly, this report should be interpreted primarily as documentation of rapid interval pseudoaneurysm formation rather than proof that pseudoaneurysm rupture directly caused the patient's death.

This case has several limitations. First, the diagnosis of left hepatic artery pseudoaneurysm was based on serial contrast-enhanced CT imaging without confirmation by dedicated CT angiography, catheter angiography, surgery, or autopsy. Second, as the patient experienced abrupt cardiovascular collapse shortly after imaging, no therapeutic intervention or additional diagnostic evaluation could be performed. Finally, as with any single case report, the findings should be interpreted as hypothesis-generating rather than establishing causality or altering current management guidelines.

## Conclusions

This case documents the rapid interval development of a left hepatic artery pseudoaneurysm within four days of presentation for presumed idiopathic acute interstitial edematous pancreatitis, as demonstrated by serial contrast-enhanced CT imaging. Unlike the majority of reported cases, the pseudoaneurysm developed in the absence of pancreatic necrosis, pseudocyst formation, or walled-off necrosis, suggesting that clinically significant vascular complications may develop earlier in the course of interstitial edematous pancreatitis than is traditionally recognized. Clinicians should maintain a high index of suspicion for vascular complications in patients who experience worsening abdominal pain, progressive abdominal distension, unexplained anemia, or unexpected clinical deterioration despite appropriate supportive management. In such patients, repeat vascular imaging, preferably with dedicated arterial-phase CT angiography when feasible, may facilitate earlier diagnosis and allow timely endovascular intervention.
